# Clinicopathological features, prognostic factor analysis, and survival nomogram of patients with double primary cancers involving lung cancer

**DOI:** 10.1002/cam4.7296

**Published:** 2024-05-21

**Authors:** Yuxuan Hao, Xiaoye Zhang, Guoyuan Cui, Xiaoying Qi, Zhongxiu Jiang, Li Yu

**Affiliations:** ^1^ Department of Oncology Shengjing Hospital of China Medical University Shenyang China; ^2^ Hematology Laboratory Shengjing Hospital of China Medical University Shenyang China

**Keywords:** Clinicopathological features, double primary cancers, lung cancer, prognostic factor analysis, survival nomogram

## Abstract

**Background:**

Although the incidence of double primary cancers (DPCs) involving lung cancer is rising, they have not been studied sufficiently. This study retrospectively analyzed the clinicopathological and prognostic characteristics of DPC patients with lung cancer and developed a survival nomogram to predict the individual OS rates.

**Methods:**

We included 103 DPC patients with lung cancer from Shengjing Hospital between 2016 and 2021. Based on the 6‐month cancer occurrence interval, the cases were categorized as synchronous DPCs (sDPCs) or metachronous DPCs (mDPCs). Furthermore, the mDPCs were subdivided based on whether the lung cancer occurred first (LCF cohort) or the other cancer occurred first (OCF cohort).

**Results:**

Among the patients, 35 (33.98%) and 68 (66.02%) had sDPCs and mDPCs, respectively. In the mDPCs cohort, 18 (26.47%) belonged to the LCF cohort and 50 (73.53%) to the OCF cohort. The most frequent primary cancer sites were the breast (27.18%), colorectum (22.33%), and urinary system (18.45%). Independent risk factors for progression‐free survival were Stage IV lung cancer (*p* = 0.008) and failure to undergo radical lung cancer surgery (*p* = 0.028). The risk factors for OS included squamous carcinoma (*p* = 0.048), Stage IV lung cancer (*p* = 0.001), single cancer resection plus drug therapy (*p* < 0.001), drug therapy alone (*p* = 0.002), failure to undergo radical lung cancer surgery (*p* = 0.014), and chemotherapy (*p* = 0.042). The median OS was 37 months, with 3‐ and 5‐year rates of 50.9% and 35.9%, respectively.

**Conclusion:**

DPCs involving lung cancer account for 1.11% of cases. The breast, colorectum, and urinary system were the most common extra‐pulmonary sites, and mDPCs were more frequent than sDPCs. Radical lung cancer surgery significantly affects prognosis, and drug therapy alone may be preferable when only one tumor is operable. The developed nomogram can accurately predict individual 3‐year and 5‐year OS rates.

## 1 INTRODUCTION

Lung cancer is one of the most prevalent cancers worldwide. According to the annual data compiled by the American Cancer Society on cancer incidence and mortality, the mortality rate for lung cancer has shown an accelerated decline in recent years. At the same time, survival rates for non‐small cell lung cancer are rapidly improving.^1^

As lung cancer survival rates improve, the late effects of the cancer become critical. The development of a new tumor is one of the serious late‐onset effects. According to the National Cancer Institute Surveillance, Epidemiology, and End Results database, the subsequent risk of developing multiple primary cancers (MPCs) is 1%–16%. The proportion of patients with the lung as the initial primary cancer site and who developed MPCs is 4%.^2^ MPCs could be attributed to the treatment of the first primary cancer, common causative factors,^3^ genetic susceptibility, environmental exposures, and a combination of factors, including gene–environment interactions and gene–gene interactions.^4^, ^5^

MPCs were first reported by Billroth in 1889. Warren and Gates proposed a refined definition of MPCs in 1932 as the presence of two or more primary malignancies of different pathological types in the body in the same or different periods after the exclusion of metastases.^6^ The International Association of Cancer Registries proposed the International Association of Cancer Registries and International Agency for Research on Cancer guidelines in 2005.^7^ These guidelines specify that if multiple cancers occur in the same organ or paired organs of the same tissue origin, they are not MPCs but rather multifocal cancers. Based on the time interval between the occurrence of the cancers, Moertel et al. classified MPCs as simultaneous MPCs (time interval ≤6 months) and heterochronic MPCs (time interval >6 months).^8^

Studies have shown that the lungs are one of the most frequently involved organs in double primary cancers (DPCs).^9^, ^10^, ^11^ The incidence of DPCs in patients with lung cancer ranges from 1.02% to 19.8%,^12^, ^13^, ^14^, ^15^ of which, the incidence reported abroad is generally higher than that reported domestically in China. In clinical practice, we have also observed an increased prevalence of DPCs involving lung cancer which have not drawn extensive attention. The low incidence of this disease makes it difficult to conduct large‐scale prospective studies; hence, retrospective studies are important. To explore the clinicopathological characteristics and prognostic and independent risk factors for DPCs involving lung cancer, 103 patients with DPCs involving lung cancer were screened for this study. A nomogram survival prediction model was constructed to provide a clinical reference for disease evaluation and prognosis assessment of such patients.

## 2 METHODS

### 2.1 Study population

There were 103 cases of DPCs involving lung cancer between January 2016 and December 2021 in the Department of Oncology at Shengjing Hospital, China Medical University. The inclusion criteria for this study were as follows: (1) two discrete lesions on imaging; (2) two tumors of different histological origins confirmed on pathological analysis; and (3) DPCs comprising lung cancer and extra‐pulmonary organ tumors. The exclusion criteria were as follows: (1) MPCs of three or more origins; (2) MPCs in the lung; and (3) metastatic cancers, recurrent cancers, and tumors without pathological diagnosis or with unclear pathological diagnosis. The follow‐ups were conducted by reviewing the outpatient and inpatient medical records or telephonically. The endpoint of the follow‐up was October 1, 2022, and the endpoint event was death. In this study, the overall survival (OS) was calculated from the diagnosis date of the second primary malignancy to the date of the last follow‐up or death. Except for Section 3.2.3 of this paper, the different definitions of OS are annotated. Progression‐free survival (PFS) focused on progression‐free survival of lung cancer after diagnosis of the second primary cancer, which was defined as the time from the diagnosis of the second primary cancer until the first progression of lung cancer or death. This study was approved by the Ethics Committee of Shengjing Hospital of China Medical University (Approval No. 2023PS838K), and the requirement for informed consent was waived considering the retrospective nature of the study.

### 2.2 Statistical analyses

Statistical analyses were performed using Statistical Product and Service Solutions, version 26.0. Kaplan–Meier curves were plotted using GraphPad Prism, version 8.0. The *t*‐test was used for inter‐group comparisons, and the chi‐square test was used for comparing categorical variables. All variables were first subjected to univariate Cox analysis, and then the variables with *p*‐values <0.05 were subjected to multivariate Cox analysis. Kaplan–Meier curves were used for survival analysis. *p*‐values < 0.05 were considered statistically significant.

### 2.3 Nomogram construction and validation methods

The nomogram model constructed using programming language R, version 4.2.2, to screen the independent risk factors for OS of the patients from the multivariate Cox proportional hazards model. The prediction accuracy of the model was assessed using the C‐index. Calibration curves were plotted to assess the predictive power of the mode.

## 3 RESULTS

### 3.1 Clinicopathological characteristics

One hundred three cases of DPCs involved lung cancer, accounted for 1.11% of lung cancer (*n* = 9288), among which, 68 were metachronous DPCs (mDPCs), and 35 were synchronous DPCs (sDPCs). In the mDPCs cohort, lung cancer occurred first in 18 patients (LCF cohort), while other cancers occurred first in 50 patients (OCF cohort). The mean age of onset in the patients was 60.05 ± 10.47 years. The mean age of the mDPCs and sDPCs cohorts was 59.48 ± 10.95 years and 61.17 ± 9.53 years, respectively. Table 1 shows the age distribution of the disease onset in both cohorts.

**TABLE 1:** Age distribution of the disease onset in the sDPCs and mDPCs cohorts.

Age (years)	sDPCs (%)	mDPCS (%)	Total (%)
<40	1 (2.86)	4 (5.88)	5 (4.85)
40–49	3 (8.57)	7 (10.29)	10 (9.71)
50–59	9 (25.71)	24 (35.29)	33 (32.04)
60–69	16 (45.71)	20 (29.41)	36 (34.95)
70–79	6 (17.14)	12 (17.65)	18 (17.48)
>79	0 (0.00)	1 (1.47)	1 (0.97)
Total	35 (100)	68 (100)	103 (100)

Abbreviations: mDPCs, metachronous double primary cancers; sDPCs, synchronous double primary cancers.

**FIGURE 1:**
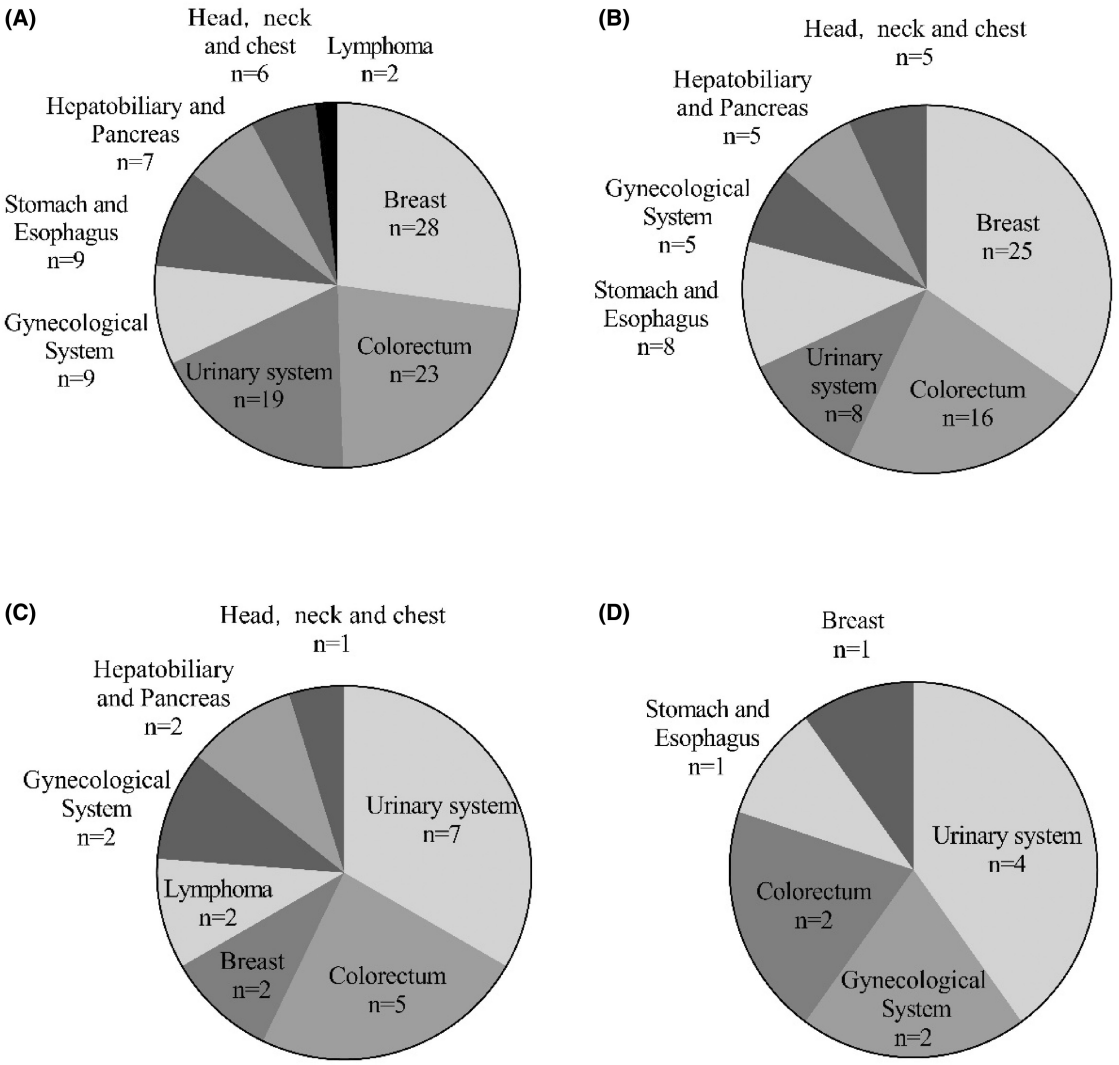
Distribution of patients with DPCs involving lung cancer. (A) Overall patients. (B) Patients with lung adenocarcinoma. (C) Patients with lung squamous cancer. (D) Patients with small cell lung cancer. Urinary system (kidney, bladder, ureter, and prostate). Gynecological system (cervix, endometrium, and ovaries). Hepatobiliary and pancreatic (liver, bile ducts, and pancreas). Head, neck, and chest (oral cavity, thyroid gland, and thymus).

**FIGURE 2:**
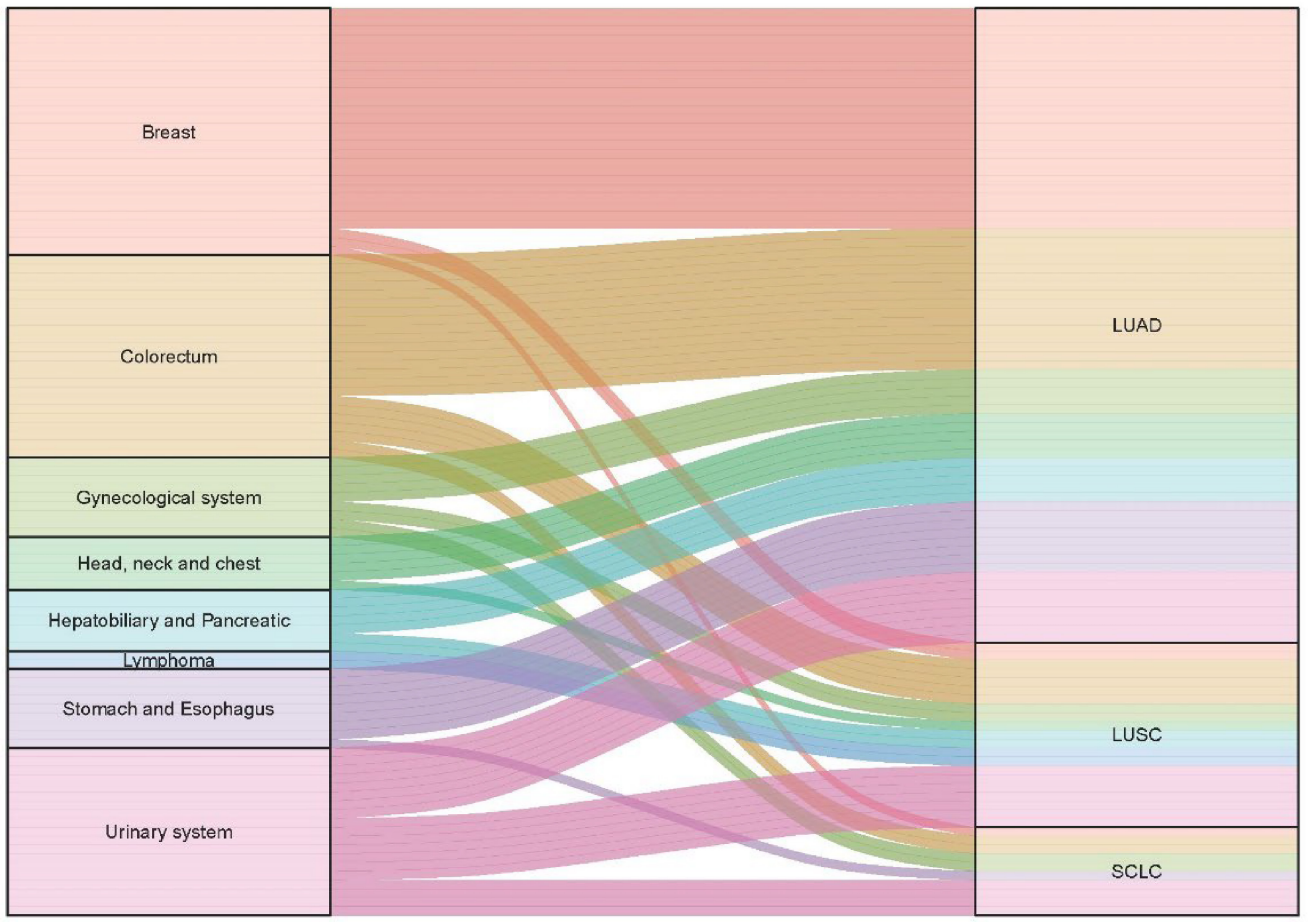
Alluvial plot showing site of DPCs involving lung cancer. LUAD Lung adenocarcinoma. LUSC, Lung squamous cell carcinoma; SCLC, Small cell lung cancer.

Figures 1 and 2 shows the tumor site distribution in the patients. The most common extra‐pulmonary sites were the breast (34.7%) and digestive system (33.3%, including stomach, esophagus, colorectum, hepatobiliary, and pancreas) in 72 patients with lung adenocarcinoma, the urinary system (33.3%) in 21 patients with lung squamous cancer and the urinary system (40%) in 10 patients with small cell lung cancer. A statistically significant difference was observed in the common extra‐pulmonary sites of development between the patients with lung adenocarcinoma and lung squamous cancer (*p* = 0.011).

We reviewed the gene mutation prevalence in the patients with NSCLC, of the 93 cases, 26 were available of the data, which was 53.85% (14/26) for EGFR, 19.23% (5/26) for RAS, 11.54% (3/26) for ALK, 7.69% (2/26) for TP53, 3.85% (1/26) for Her‐2, and 3.85% (1/26) for RET.

Table 2 shows that 70.87% of the second primary cancer developed within 4 years from the diagnosis of the first primary cancer. The probability of developing a second primary cancer decrease along with time. The median time interval for patients in the mDPCs cohort was 37.5 months, with a mean time interval of 68.93 ± 7.68 months.

**TABLE 2:** Time interval between two primary cancers.

Metachronous interval time (years)	Number	Percentage
0–0.5	35	33.98%
0.5–2	21	20.39%
2–4	17	16.50%
4–6	4	3.88%
6–8	6	5.83%
8–10	8	7.77%
10–12	4	3.88%
12–14	1	0.97%
14–16	4	3.88%
16–18	2	1.94%
>18	1	0.97%
Total	103	100%

The comparison of the clinicopathological characteristics of the sDPCs and mDPCs cohorts revealed that patients in the former were commonly treated with double cancer resection surgery, while those in the latter were commonly treated with single cancer resection surgery plus drug therapy (*p* = 0.001). Moreover, a higher proportion of patients underwent chemotherapy in the sDPCs group than in the mDPCs group (*p* = 0.017) (Table 3).

**TABLE 3:** Clinicopathological characteristics in the sDPCs and mDPCs cohorts.

	Total (*n* = 103)	sDPCs (*n* = 35)	mDPCs (*n* = 68)	*p*
Age(years)				0.167
<60	48 (46.6)	13 (37.1)	35 (51.5)	
≥60	55 (53.4)	22 (62.9)	33 (48.5)	
Sex				0.891
Male	51 (49.5)	17 (48.6)	34 (50)	
Female	52 (50.5)	18 (51.4)	34 (50)	
CEA				0.694
Negative	65 (63.1)	23 (65.7)	42 (61.8)	
Positive	38 (36.9)	12 (34.3)	26 (38.2)	
LC pathology				0.6
Squamous cancer	21 (20.4)	7 (20)	14 (20.6)	
Adenocarcinoma	72 (69.9)	26 (74.3)	46 (67.6)	
Small cell cancer	10 (9.7)	2 (5.7)	8 (11.8)	
LC stage				0.590
I + II	61 (59.2)	22 (62.9)	39 (57.4)	
III + IV	42 (40.8)	13 (37.1)	29 (42.6)	
LC Ki‐67				0.561
<30%	6 (5.8)	1 (2.9)	5 (7.4)	
≥30%	14 (13.6)	4 (11.4)	10 (14.7)	
Unknown	83 (80.6)	30 (85.7)	53 (77.9)	
LC differentiation				0.865
High	13 (12.6)	5 (14.3)	8 (11.8)	
Medium, medium‐high	7 (6.8)	2 (5.7)	5 (7.4)	
Low, medium‐low	25 (24.3)	7 (20)	18 (26.5)	
Unknown	58 (56.3)	21 (60)	37 (54.4)	
Treatment				0.001
Double resection	38 (36.9)	16 (45.7)	22 (32.3)	
Single resection+drug	43 (41.8)	6 (17.1)	37 (54.4)	
Drug	22 (21.3)	13 (37.1)	9 (13.2)	
LC surgery				0.2
Yes	44 (42.7)	18 (51.4)	26 (38.2)	
No	59 (57.3)	17 (48.7)	42 (61.8)	
Chemotherapy				0.017
Yes	73 (70.9)	30 (85.7)	43 (63.2)	
No	30 (29.1)	5 (14.3)	25 (36.8)	
Radiotherapy				0.3
Yes	26 (25.2)	11 (31.4)	15 (22.1)	
No	77 (74.8)	24 (68.6)	53 (77.9)	
Immune/targeted therapy				0.492
Yes	34 (33.0)	10 (28.6)	24 (35.3)	
No	69 (67.0)	25 (71.4)	44 (64.7)	

Abbreviations: LC, Lung cancer; mDPCs, metachronous double primary cancers; sDPCs, synchronous double primary cancers.

The results showed that the mean interval between the occurrences of cancers in the LCF cohort was shorter than that in the OCF cohort (42.72 ± 10.31 months vs. 78.36 ± 9.47 months; *p* = 0.019). The proportion of patients with Stage I and II lung cancer was significantly higher in the LCF cohort than in the OCF cohort (*p* = 0.041). Patients in the LCF cohort were more likely to undergo double cancer resection, while those in the OCF cohort were more likely to undergo single cancer resection plus drug therapy (*p* = 0.044). The proportion of patients who underwent radical lung cancer surgery was significantly higher in the LCF cohort than in the OCF cohort (*p* = 0.001). The proportion of patients who underwent radiotherapy was higher in the OCF cohort than in the LCF cohort (*p* = 0.049) (Table 4).

**TABLE 4:** Clinicopathological characteristics in the LCF and OCF cohorts.

	Total (*n* = 68)	LCF (*n* = 18)	OCF (*n* = 50)	*p*
Age(years)				0.073
<60	35 (51.5)	6 (33.3)	29 (58)	
≥60	33 (48.5)	12 (66.7)	21 (42)	
Sex				0.272
Male	34 (50)	11 (61.1)	23 (46)	
Female	34 (50)	7 (38.9)	27 (54)	
CEA				0.947
Negative	42 (61.8)	11 (61.1)	31 (62)	
Positive	26 (38.2)	7 (38.9)	19 (38)	
LC pathology				0.875
Squamous cancer	14 (20.6)	3 (16.7)	11 (22)	
Adenocarcinoma	46 (67.6)	13 (72.2)	33 (66)	
Small cell cancer	8 (11.8)	2 (11.1)	6 (12)	
Interval between onset				0.019
<37.5 moths	33 (48.5)	13 (72.2)	20 (40)	
≥37.5 moths	35 (51.5)	5 (27.8)	30 (60)	
LC Stage				0.041
I + II	39 (57.4)	14 (77.8)	25 (50)	
III + IV	29 (42.6)	4 (22.2)	25 (50)	
Treatment				0.044
Double resection	22 (32.4)	10 (55.6)	12 (24)	
Single resection+drug	37 (54.4)	7 (38.9)	30 (60)	
Drug	9 (13.2)	1 (5.6)	8 (16)	
LC surgery				0.001
Yes	26 (38.2)	13 (72.2)	13 (26)	
No	42 (61.8)	5 (27.8)	37 (74)	
Chemotherapy				0.356
Yes	43 (63.2)	13 (72.2)	30 (60)	
No	25 (36.8)	5 (27.8)	20 (40)	
Radiotherapy				0.049
Yes	15 (22.1)	1 (5.6)	14 (28)	
No	53 (77.9)	17 (94.4)	36 (72)	
Immune/Targeted therapy				0.343
Yes	24 (35.3)	10 (55.6)	16 (32)	
No	44 (64.7)	8 (44.4)	34 (68)	

Abbreviations: LC, lung cancer; LCF, lung cancer first; OCF, other cancer first.

### 3.2 Prognostic analysis

#### 3.2.1 Prognostic analysis for PFS

According to the results of univariate and multivariate Cox models, Stage IV lung cancer (*p* = 0.008) and failure to undergo radical lung cancer surgery (*p* = 0.028) were significantly associated with poor progression‐free survival (PFS) in patients with DPCs involving lung cancer (Table 5).

**TABLE 5:** Univariate and multivariate Cox models for PFS.

	Univariate		Multivariate	
	HR (95% CI)	*p*	HR (95% CI)	*p*
Age (years)				
<60	1			
≥60	1.008 (0.603–1.684)	0.977		
Sex				
Female	1			
Male	1.346 (0.804–2.253)	0.259		
CEA				
Negative	1		1	
Positive	1.759 (1.047–2.956)	0.033	1.218 (0.671–2.213)	0.516
LC pathology				
Adenocarcinoma	1	0.617		
Squamous cancer	1.349 (0.728–2.502)	0.342		
Small cell cancer	1.223 (0.436–3.429)	0.702		
LC stage				
I	1	<0.001	1	0.034
II	1.439 (0.642–3.227)	0.377	1.047 (0.450–2.433)	0.915
III	2.349 (1.233–4.474)	0.009	1.119 (0.499–2.509)	0.785
IV	8.755 (3.619–21.18)	<0.001	3.867 (1.434–10.427)	0.008
LC Ki‐67				
<30%	1			
≥30%	1.611 (0.321–4.20)	0.820		
LC differentiation				
High	1	0.301		
Medium, medium‐high	0.436 (0.090–2.111)	0.302		
Low, medium‐low	1.358 (0.554–3.329)	0.503		
Treatment				
Double resection	1	0.003	1	0.478
Single resection+drug	2.688 (1.490–4.847)	0.001	0.508 (0.123–2.109)	0.351
Drug	2.600 (1.203–5.218)	0.015	0.371 (0.073–1.889)	0.233
LC surgery				
No	1		1	
Yes	0.286 (0.160–0.509)	<0.001	0.203 (0.049–0.845)	0.028
Chemotherapy				
No	1			
Yes	1.659 (0.918–2.998)	0.094		
Radiotherapy				
No	1			
Yes	1.281 (0.708–2.317)	0.413		
Immune/Targeted therapy				
No	1		1	
Yes	2.181 (1.305–3.646)	0.003	1.549 (0.795–3.019)	0.198

Abbreviations: LC, lung cancer; PFS, progression‐free survival.

#### 3.2.2 Prognostic analysis for OS (OS started from diagnosis of the second primary cancer)

Squamous carcinoma (*p* = 0.048), Stage IV lung cancer (*p* = 0.001), single cancer resection plus drug therapy (*p* < 0.001), drug therapy alone (*p* = 0.002), failure to undergo radical lung cancer surgery (*p* = 0.014), and chemotherapy (*p* = 0.042) were associated with poor OS in patients with DPCs involving lung cancer (Table 6).

**TABLE 6:** Univariate and multivariate Cox models for OS.

	Univariate		Multivariate	
	HR (95% CI)	*p*	HR (95% CI)	*p*
Age (years)				
<60	1			
≥60	0.932 (0.554–1.567)	0.789		
Sex				
Female	1		1	
Male	2.106 (1.235–3.591)	0.006	1.241 (0.648–2.378)	0.515
CEA				
Negative	1		1	
Positive	1.792 (1.046–3.071)	0.034	1.171 (0.655–2.096)	0.594
Time interval				
sDPCs	1			
mDPCs	1.123 (0.635–1.988)	0.689		
LC pathology				
Adenocarcinoma	1	0.001	1	0.140
Squamous cancer	2.815 (1.521–5.208)	0.001	2.117 (1.006–4.455)	0.048
Small cell cancer	2.571 (1.134–5.830)	0.024	1.412 (0.543–3.672)	0.480
LC stage				
I	1	<0.001	1	0.008
II	1.374 (0.575–3.286)	0.475	1.050 (0.388–2.840)	0.923
III	3.542 (1.823–6.885)	<0.001	1.651 (0.733–3.723)	0.226
IV	9.068 (3.833–21.455)	<0.001	5.942 (2.037–17.030)	0.001
LC Ki‐67				
<30%	1			
≥30%	2.259 (1.225–79.085)	0.031		
LC differentiation				
High	1	0.015		
Medium, medium‐high	2.352 (0.389–14.225)	0.352		
Low, medium‐low	6.787 (1.570–29.342)	0.01		
Treatment				
Double resection	1	<0.001	1	0.002
Single resection+drug	4.095 (2.080–8.061)	<0.001	12.401 (3.111–49.434)	<0.001
Drug	3.798 (1.770–8.150)	0.001	11.605 (2.466–54.606)	0.002
LC surgery				
No	1		1	
Yes	0.316 (0.174–0.573)	<0.001	0.19 (0.051–0.711)	0.014
Chemotherapy				
No	1		1	
Yes	2.383 (1.198–4.739)	0.013	2.251 (1.029–4.923)	0.042
Radiotherapy				
No	1			
Yes	1.124 (0.642–1.967)	0.684		
Immune/Targeted therapy				
No	1		1	
Yes	2.082 (1.221–3.549)	0.007	1.159 (0.616–2.179)	0.648

Abbreviations: LC, lung cancer; OS, overall survival.

Stage IV lung cancer (*p* = 0.008), single cancer resection plus drug therapy (*p* = 0.002), drug therapy alone (*p* = 0.002), failure to undergo radical lung cancer surgery (*p* = 0.014), and chemotherapy (*p* = 0.019) were associated with poor OS in the mDPCs cohort. There was no significant difference in the OS between LCF and OCF patients (*p* = 0.405) (Table 7). We have collected the data on the prognosis of the first primary cancers in mDPCs (*n* = 68), of which, 24 achieved “clinical cure”, defined as “the absence of progression, recurrence, or metastasis within 5 years after therapeutic courses,” and 44 did not reach the standard of a clinical cure. Whether or not the first tumor was cured had no effect on the prognosis of the second tumor (*p* = 0.686).

**TABLE 7:** Univariate and multivariate Cox models for OS in mDPCs cohort.

	Univariate		Multivariate	
	HR (95% CI)	*p*	HR (95% CI)	*p*
Age (years)				
<60	1			
≥60	0.957 (0.514–1.783)	0.890		
Sex				
Female	1			
Male	1.854 (0.983–3.498)	0.057		
CEA				
Negative	1			
Positive	1.873 (1–3.510)	0.05		
Time interval				
<37.5	1			
≥37.5	0.785 (0.419–1.471)	0.450		
Order of LC				
LCF	1			
OCF	1.390 (0.640–3.023)	0.405		
LC pathology				
Adenocarcinoma	1	0.001	1	0.154
Squamous cancer	2.926 (1.372–6.241)	0.005	2.097 (0.921–4.773)	0.078
Small cell cancer	3.917 (1.652–9.288)	0.002	1.936 (0.669–5.603)	0.223
LC stage				
I	1	<0.001	1	0.015
II	0.878 (0.274–2.819)	0.827	0.458 (0.122–1.717)	0.247
III	4.160 (1.823–9.495)	0.001	1.095 (0.391–3.065)	0.862
IV	9.228 (3.467–24.560)	<0.001	4.399 (1.467–13.193)	0.008
Treatment				
Double resection	1	<0.001	1	0.006
Single resection+drug	6.076 (2.313–15.963)	<0.001	22.997 (3.312–159.697)	0.002
Drug	9.265 (3.039–28.247)	<0.001	25.613 (3.194–205.378)	0.002
LC surgery				
No	1		1	
Yes	0.219 (0.096–0.501)	<0.001	0.142 (0.028–0.729)	0.019
Chemotherapy				
No	1		1	
Yes	2.592 (1.223–5.491)	0.013	2.176 (0.889–5.324)	0.089
Radiotherapy				
No	1			
Yes	1.209 (0.589–2.479)	0.605		
Immune/Targeted therapy				
No	1		1	
Yes	2.304 (1.225–4.333)	0.01	1.315 (0.628–2.753)	0.467

Abbreviations: LC, lung cancer; OS, overall survival.

**FIGURE 3:**
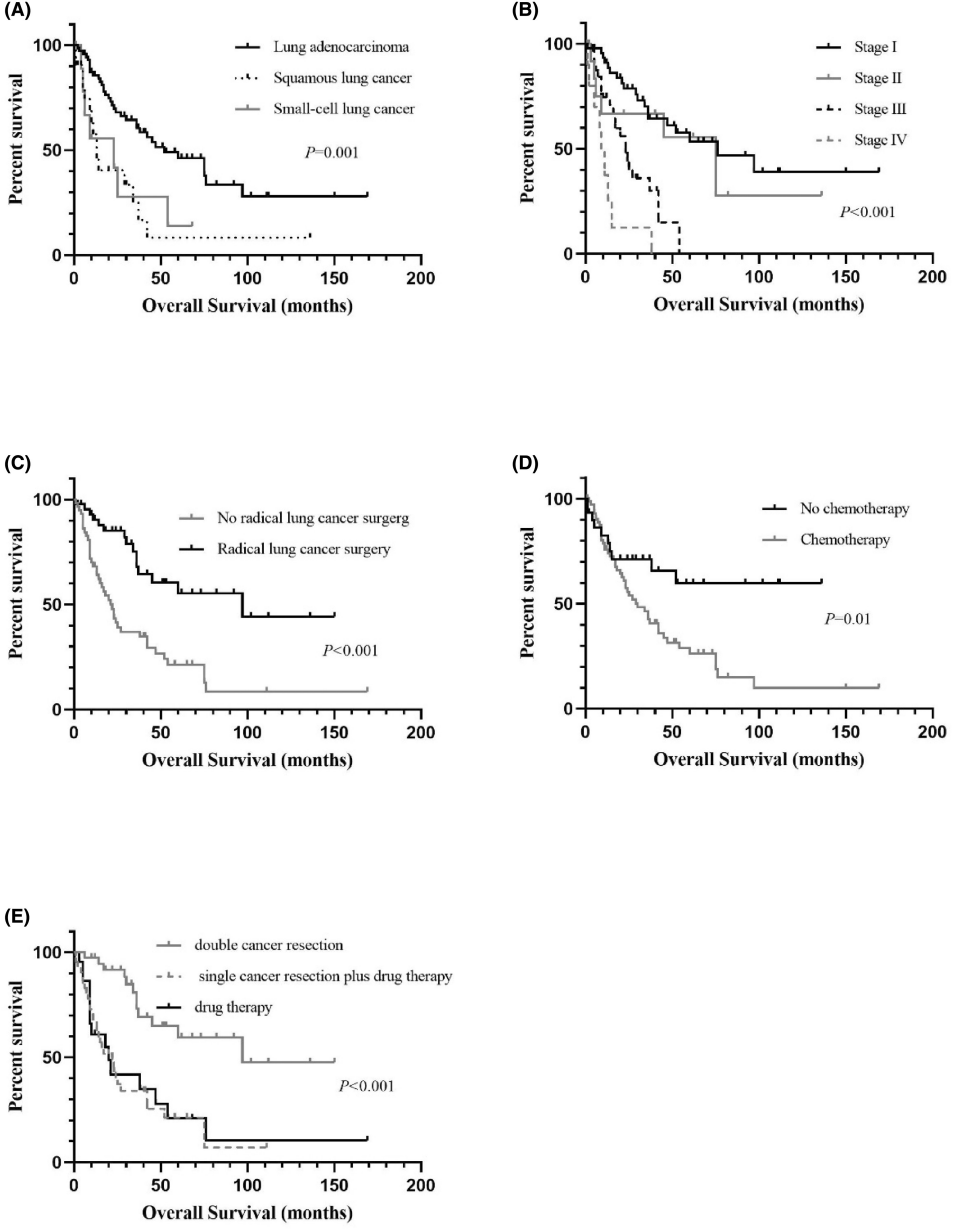
Kaplan–Meier survival curves of overall survival based on pathology (A), staging (B), surgery (C), chemotherapy (D), and treatment (E).

The median OS for patients with DPCs involving lung cancer was 37 months, with a mean survival time of 64.09 ± 7.88 months. The 3‐year and 5‐year OS rates were 50.9% and 35.9%, respectively. Table 8 shows the survival of the patients in different subgroups. Figure 3 shows the Kaplan–Meier curves of the patients in different subgroups.

**TABLE 8:** Mean OS and OS rates of sub‐groups.

	Mean OS (m)	OS rates (%)		*p*
		3‐year	5‐year	
LC pathology				0.001
Adenocarcinoma	75.98 ± 9.78	60.5	46.2	
Squamous cancer	27.83 ± 9.34	25.2	8.4	
Small cell cancer	26.27 ± 8.07	27.8	13.9	
LC stage				<0.001
I	92.11 ± 12.36	53.6	39.1	
II	65.53 ± 16.08	66.7	55.6	
III	26.36 ± 3.28	36	0	
IV	12.13 ± 3.83	12.5	0	
Treatment				<0.001
Double resection	96.83 ± 11.65	73.2	59.6	
Single resection+drug	32.90 ± 5.66	34	21.3	
Drug	42.0 ± 12.78	41.8	20.9	
LC surgery				<0.001
No	39.55 ± 7.36	37	21.4	
Yes	90.54 ± 11.39	68	55.4	
Chemotherapy				0.01
No	88.84 ± 11.88	71.3	59.8	
Yes	47.72 ± 7.30	42.6	26.3	

Abbreviations: LC, lung cancer; OS, overall survival.

#### 3.2.3 Prognosis of sDPCs versus mDPCs

**FIGURE 4:**
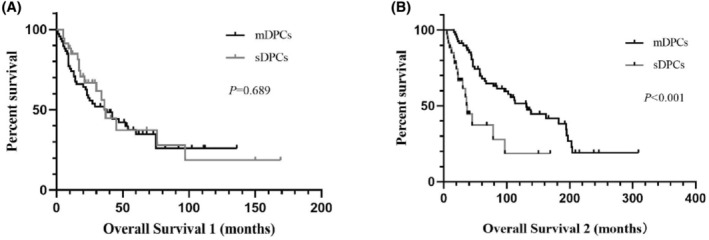
Kaplan–Meier survival curves for overall survival 1 and overall survival 2 of sDPCs versus mDPCs. *sDPCs* synchronous double primary cancers. *mDPCs* metachronous double primary cancers. *Overall survival 1* the time interval between the first primary cancer diagnosis and patient's death (A). *Overall survival 2*: the time interval between the second primary cancer diagnosis and patient's death (B).

The patients with sDPCs have a shorter OS (OS started from diagnosis of the first primary cancer) than patients with mDPCs (*p* < 0.001). Whereas there was no statistically significant difference between the OS (OS started from diagnosis of the second primary cancer) of mDPCs and sDPCs (*p* = 0.689) (Figure 4).

### 3.3 Survival nomogram

**FIGURE 5:**
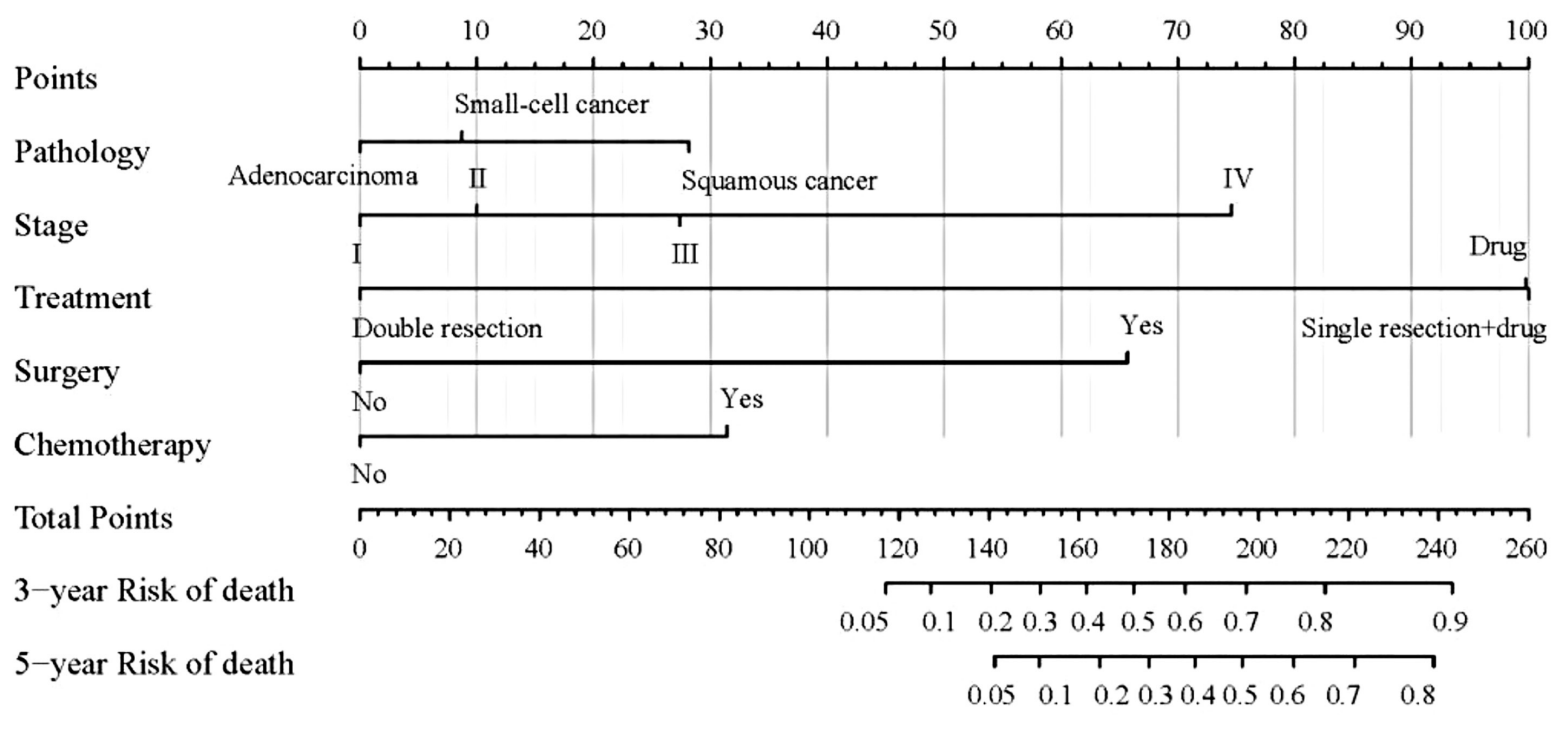
Nomogram to predict 3‐ and 5‐year OS rates of the patients with DPCs involving lung cancer. The factors of LC pathology, LC stage, LC surgery, chemotherapy and treatment were included in the model. DPCs, double primary cancers; LC, lung cancer; OS, overall survival.

**FIGURE 6:**
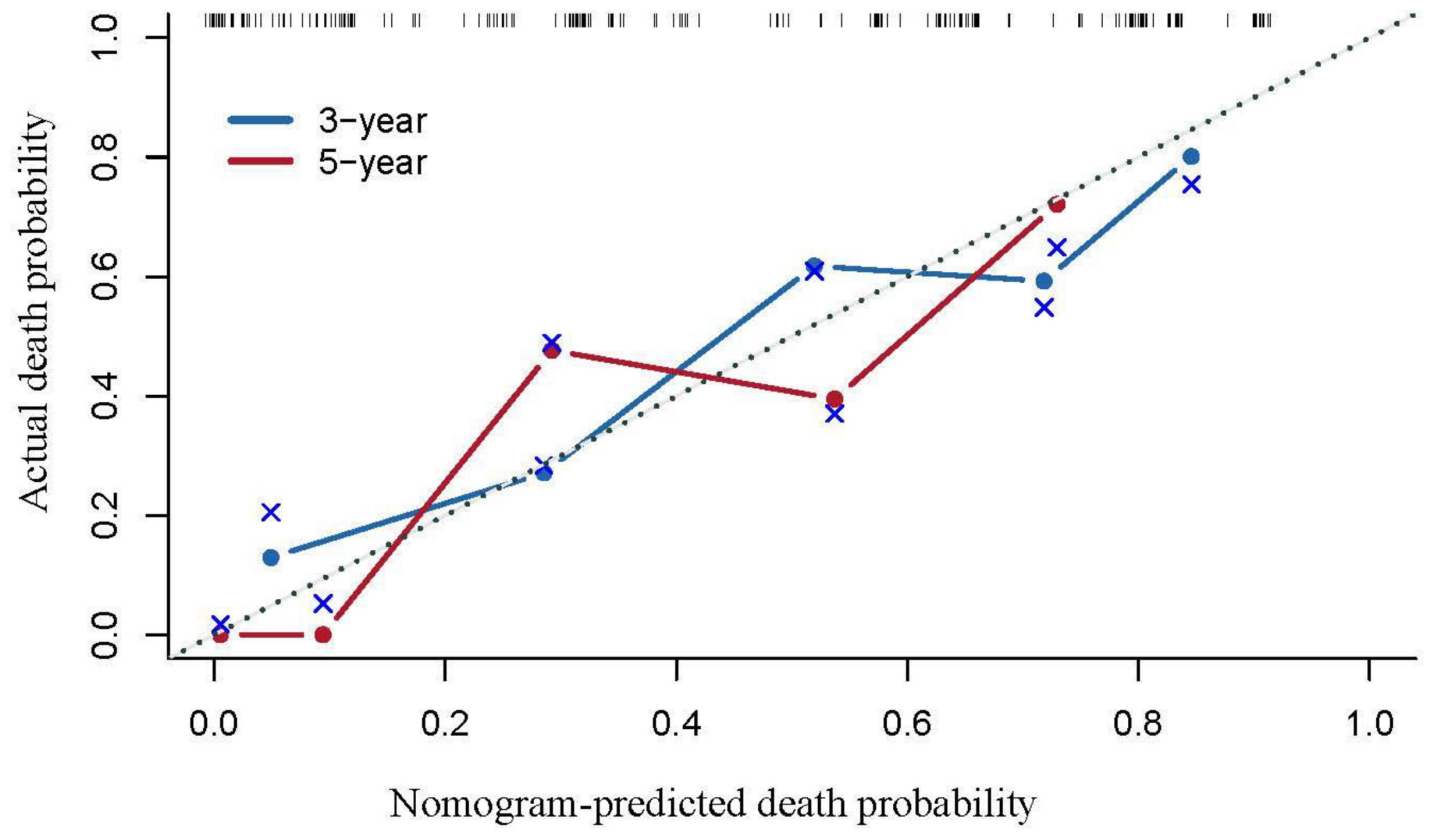
Calibration curve of the survival nomogram.

The independent risk factors for OS, including lung cancer pathological type, lung cancer stage, treatment, previous radical lung cancer surgery, and chemotherapy, were used to construct a nomogram prediction model using R, version 4.2.2 (Figure 5). Non‐collinearity by the variance inflation factor (VIF) test was confirmed for treatment modality (VIF = 3.031), presence/absence of previous radical lung cancer surgery (VIF = 3.086), and chemotherapy (VIF = 1.053). The model was internally validated by the Bootstrap self‐sampling method with self‐sampling number B = 1000. The C‐index value was 0.771 (95% confidence interval: 0.712–0.83), indicating moderate accuracy, and the calibration curve was close to the standard curve variation and showed a good prediction performance value (Figure 6).

## 4 DISCUSSION

The incidence of MPCs is reportedly 0.26%–9%.^16^, ^17^, ^18^, ^19^ The incidence of DPCs involving lung cancer was 1.02–19.8% in patients with lung cancer. The incidence of DPCs in patients with lung cancer in this study was 1.11%, which was similar to that reported previously. However, because some patients with incomplete clinical data were excluded, the incidence of DPCs involving lung cancer in this study may have been slightly less than the actual incidence. The median interval between the onset of the two cancers in this study was 37.5 months, which is roughly the same as the 38 months reported by Vadgaonkar et al.^17^ The number of patients with Stage I and II lung cancer was higher in the LCF cohort than in the OCF cohort in this study, which is consistent with previous studies where the OCF group had a worse prognosis compared with the LCF group.^20^ The interval between the onset of the two cancers was longer in the OCF cohort than in the LCF cohort, which corroborates the result of previous study.^21^ With improved medical care, many lung cancers are diagnosed and treated at early stages. The Chinese Lung Cancer Epidemiology Survey showed that 57.2% of non‐small cell lung cancer patients were first diagnosed with Stage 0 to Stage III, and 57.6% of small cell lung cancer patients were first diagnosed with limited stages.^22^ In our study, 59.2% of patients were diagnosed at Stage I/II lung cancer. In actual clinical practice, we also found that a growing number of patients with lung ground‐glass nodules opted for surgical resection. Part of patients in the OCF cohort may not undergo radical lung cancer surgery due to their physical status or pessimistic attitude for the treatment. The combination of the above reasons may lead to our conclusion that the patients in the LCF cohort were more likely to undergo double cancer resection, while those in the OCF cohort were more likely to undergo single cancer resection plus drug therapy.

The study by Etiz et al. on MPCs showed that the first primary cancer sites were head and neck (22%), breast (20%), and gastrointestinal system (20%), and the second primary cancer sites were gastrointestinal tract (22%), lungs (19%), and gynecological system (15%).^16^ The study by Liu et al. on MPCs involving lung cancer showed that the upper respiratory tract, gastrointestinal tract, and colorectum are the most likely cancer sites.^23^ Feng et al. showed that the top three preferred cancers in heterochronic MPCs were colorectal, head and neck, and lung cancers, while the top three preferred cancers in simultaneous MPCs were lung, colorectal, and breast cancers.^24^ In this study, the top three extra‐pulmonary sites in patients with DPCs involving lung cancer were the breast (27.18%), colorectum (22.33%), and urinary system (18.45%). The top two extra‐pulmonary sites in patients with lung adenocarcinoma were the breast (34.7%) and digestive system (33.3%), including the colorectum, stomach, and esophagus. The most common extra‐pulmonary site in patients with lung squamous cancer was the urinary system (33.3%), including the kidneys, bladder, ureters, prostate. The results of this study were almost similar to those of the aforementioned studies. Patients with lung adenocarcinoma and lung squamous cancer had different extra‐pulmonary preference sites, and the differences were statistically significant. This could be attributed to various factors. First, MPCs tend to develop in organs with similar histological types. Lung adenocarcinoma tends to occur in combination with breast and gastrointestinal tract cancers, both of which are composed of glands with secretory functions, and the pathological type of the cancer is mostly adenocarcinoma. Squamous carcinoma of the lung tends to occur in combination with urological tumors, as the lower ureter, bladder, and urethra are covered with squamous epithelium. Second, it may be associated with common genetic mutations. For example, *HER‐2* amplification and mutation can occur in lung, breast, and gastric cancers, and the *HER‐2* gene mutation is more common in lung adenocarcinoma than in lung squamous cancer.^25^, ^26^ *EGFR* and *c‐MET* gene mutations can occur in both lung and gastric cancers, and the abnormal expression of *c‐MET* is mainly reflected in non‐small cell lung cancer.^27^ Mutated genes common to both lung and colorectal cancers include *BRAF* and *KRAS*. Recently, it has been shown that the *ALK* and *ROS1* genes driving lung cancer are also present in colorectal cancer.^28^

Our study concluded that squamous carcinoma, Stage IV lung cancer, resection of single cancer plus drug therapy or drug therapy alone, failure to undergo radical lung cancer surgery, and chemotherapy were associated with poor OS in patients with DPCs involving lung cancer. Several previous studies have concluded that patients with higher stages of lung cancer have a worse prognosis.^12^, ^29^ The study by Ventura et al. revealed a poor prognosis for MPCs of lung squamous carcinoma, which corroborates the results of this study.^15^ The prognosis of patients treated with single cancer resection plus drug therapy or drug therapy alone, without radical lung cancer surgery and chemotherapy, is poor, which could be attributed mainly to the following two points. First, patients who can undergo double cancer surgery or radical lung cancer surgery without chemotherapy have earlier cancer stages and better biological behavior. Second, since the general status of patients who can tolerate surgical treatment is better, they may have a better prognosis.

This study showed that failure to undergo radical lung cancer surgery was an independent risk factor for PFS and OS in patients with DPCs involving lung cancer with a high hazard ratio (HR). Therefore, we believe that the prognosis of lung cancer plays a major role in the overall prognosis of patients with DPCs involving lung cancer.

According to the HR derived from the multivariate Cox model, the probability of death was 12.401 times higher in patients treated with single cancer resection plus drug therapy than in patients treated with double cancer resection. The probability of death was 11.605 times higher in patients treated with drug therapy alone than in patients treated with double cancer resection. Therefore, in patients with DPCs involving lung cancer, we believe that double cancer resection is better for a good prognosis. In contrast, in cases where only one tumor is eligible for surgery, the prognosis does not differ much between single cancer surgery and drug therapy alone. The results of the survival analysis in this study corroborate these findings. The mean survival times were lower for patients treated with single cancer resection plus drug therapy than for patients treated with drug therapy alone. This could be related to the damage caused by surgery, postoperative decrease in immunity, and various postoperative complications. We believe that drug therapy alone could be a better choice than surgery plus drug therapy for single cancer when only one tumor is eligible for surgery. Regarding the definition of OS in DPCs, it was categorized into two types based on previous studies as OS (OS started from diagnosis of the first primary cancer) and OS (OS started from diagnosis of the second primary cancer). We statistically analyzed both definitions of OS and came to the same conclusion as the previous study that the OS (OS started from diagnosis of the first primary cancer) of mDPCs was significantly better than that of sDPCs, whereas there was no statistically significant difference between the OS(OS started from diagnosis of the second primary cancer) of mDPCs and sDPCs.^30^

Patients with DPCs involving lung cancer are highly individualized, and no standard treatment protocol exists. Surgical resection is recommended in cases where the primary cancer is suitable for radical surgery. Radiotherapy, chemotherapy, targeted therapy, and immunotherapy can be chosen for patients with advanced cancer stages that cannot be operated. If there are common effective drugs for both cancers, then they should be chosen. The case report by Okamoto et al. showed that platinum‐containing anticancer drugs, such as carboplatin, were effective in treating hepatocellular carcinoma combined with lung cancer in a concurrent double primary tumor.^31^ When it is not possible to treat both tumors with the same treatment, priority is given to treating tumors with recurrent metastases, poor differentiation, or worse clinical symptoms. The advent of immune checkpoint inhibitors also offers new options for treating patients with DPCs involving lung cancer. There have been several case reports of successful combination therapy with immune checkpoint inhibitors for DPCs, including nabrituzumab, carrilizumab, pablizumab, and atelelizumab.^32^, ^33^, ^34^, ^35^ The combination of radiotherapy, chemotherapy, and low‐toxicity immune and targeted agents has shown promise in the individualized treatment of patients with inoperable DPCs.^34^, ^36^

The development of MPCs is associated with various factors.^10^, ^37^ Lifestyle behaviors, including smoking and excessive alcohol consumption, contribute to the development of MPCs. Moreover, common environmental exposures, including environmental pollution, occupational exposures, and infections, contribute to the development of subsequent cancers. Treatments for first primary cancers, such as chemotherapy, radiotherapy, endocrine therapy, and hematopoietic stem cell transplantation, can increase the risk of second primary cancers.^38^, ^39^, ^40^ Patients receiving chemotherapy are known to be at increased risk of developing secondary tumors, and the challenge ahead is to minimize the mutagenic effects of these drugs by adjusting their cumulative dose without compromising their therapeutic benefit.^41^ The mechanisms by which genetic susceptibility contributes to the increased risk of developing second primary tumors are complex. It is mostly characterized by family history and familial syndromes, including hereditary breast and ovarian cancers, hereditary nonpolyposis colorectal cancer, or Lynch syndrome.^42^, ^43^, ^44^ Genetic mutations are associated with an increased risk of developing second primary cancers. For example, *BRCA1* and *BRCA2* mutations are associated with an increased risk of second primary breast or ovarian cancer.^45^ Tumor protein‐53 (TP53) germline pathogenic variants are associated with a variety of primary cancers, including soft tissue and bone sarcomas, breast cancer, brain tumors, adrenocortical carcinoma, and leukemia; therefore, patients with TP53 germline pathogenic variants should undergo annual whole‐body medical check‐ups for the secondary prevention of MPCs.^46^

The diagnosis and treatment of MPCs mostly rely on the treatment experience of the clinical, pathological, and imaging physicians. Positron emission tomography/computed tomography is effective in identifying MPCs, which helps in the early identification and management of MPCs.^47^ Several studies have shown that molecular diagnostics have promising applications in the diagnosis of DPCs. Hatakeyama et al. showed that whole‐exome sequencing detects differences in the origin of multiple tumors from the same patient by analyzing the concordance of somatic mutations in DPCs.^48^ These gene mutations mentioned above associated with the development of multiple tumors could be the key to treating MPCs. However, there are some limitations of our study such as lack of data on family history, smoking history, cause of death and incomplete data on gene mutations, due to the retrospective character of this study, this information is no longer available. Larger size studies with comprehensive data of double primary cancers involving lung cancers are needed to validate the existing findings. Clinical databases of MPCs should be established in the future, comprising statistics on their risk factors and prevalent populations, to review and optimize previous treatment protocols and guide the comprehensive treatment of patients with MPCs.

## 5 CONCLUSION

DPCs involving lung cancer account for 1.11% of cases. The breast, colorectum, and urinary system were the most common extra‐pulmonary sites, and mDPCs were more frequent than sDPCs. Radical lung cancer surgery significantly affects prognosis, and drug therapy alone may be preferable when only one tumor is operable. The developed nomogram can accurately predict individual 3‐year and 5‐year OS rates.

## AUTHOR CONTRIBUTIONS

**Yuxuan Hao:** Data curation (equal); formal analysis (equal); writing – original draft (lead). **Xiaoye Zhang:** Conceptualization (lead); writing – review and editing (equal). **Guoyuan Cui:** Data curation (equal); formal analysis (equal); writing – review and editing (equal). **Xiaoying Qi:** Data curation (equal); formal analysis (equal); visualization (equal). **Zhongxiu Jiang:** Data curation (equal); formal analysis (equal); visualization (equal). **Li Yu:** Conceptualization (equal); writing – review and editing (lead).

## FUNDING INFORMATION

This research did not receive any specific grant from funding agencies in the public, commercial, or not‐for‐profit sectors.

## CONFLICT OF INTEREST STATEMENT

The authors declare that they have no known competing financial interests or personal relationships that could have appeared to influence the work reported in this paper.

## ETHICS STATEMENT

The study was approved by the Ethics Committee of Shengjing Hospital of China Medical University‐agreement NO. 2023PS838K, which waived the requirement for informed consent in view of the retrospective nature of the study.

## Data Availability

The datasets used and/or analyzed during the current study are available from the corresponding author on reasonable request.
